# Supplementary material for: Health Effects of Red Wine Consumption: A Narrative Review of an Issue That Still Deserves Debate
Source: Nutrients. 2023 Apr 16;15(8):1921. doi: 10.3390/nu15081921 (PMC10146095; doi:10.3390/nu15081921)
Supplement: Supplementary file 1 [file nutrients-15-01921-s001.zip › Table S1 - Mean features of all papers (1).pdf]

ref.	First author	year	Type of Study	Type of Wine	Subjects	Patients	Age range	Total number of participants	Wine consumption duration (days)	Control	Dosage (mL)	Funding
10	Agewall S	2000	RCT CO	RW	M+F	H	27 - 35	12	acute	DRW	250	Swedish Medical Research Council, Swedish Medical Society
11	Caccetta RA	2000	RCT	RW	M	H	40 - 63	12	acute	DRW, PSRW, W	5/kg BW	The National Heart Foundation of Australia and the Medical Research Foundation of Royal Perth Hospital
12	Senault C	2000	RCT CO	RW	M	H	18 - 35	56	14	DRW, ET	30g of ET	ONIVINS and INSERM, Paris, France.
13	Caccetta R	2001	RCT	RW	M	H, smokers	25 - 71	18	14	DRW, WW	375	Australian Grape Wine Research and Development Corporation and the Medical Research Foundation of Royal Perth Hospital.
14	Ceriello A	2001	RCT	RW	M+F	T2DM	50 - 60	20	7	W	300	Not declared
15	De Vries JH	2001	RCT	RW	M	H	20 - 35	12	4	black tea, fried onions	750	Netherlands Heart Foundation
16	Van der Gaag MS	2001	RCT CO	RW	M+F	H	44 - 59	11	21	beer, spirits, W	4 glasses (40 g ET)	Not declared
17	Mansvelt EP	2002	RCT	RW	M+F	H	25-56	13	28	WW	ET: 23 g for F, 32 g for M	Not declared
18	Foppa M	2002	RCT CO	RW	M+F	HPN, obese	35 - 65	13	acute	W	250 (23 g ET)	Not declared
19	Watzl B	2002	RCT	RW	M	H	28 - 32	6	1	DRW, red grape juice	500 ml RW (12 % ET), a 12 % ET dilution, DRW, and red grape juice	Federal Ministry of Consumer Protection, Food, and Agriculture, Germany.
20	Mezzano D	2003	RCT	RW	M	H	19 - 25	21	90	MD	240 (23.2 g ET)	University of Chile
21	Pignatelli P	2003	RCT	RW	M+F	H	35 - 50	24	14	WW	300	Not declared

22	Watzl B	2003	RCT	RW	M	H	25 - 35	24	14	ET	500 (12 % ET), 12 % ET, DRW, and red grape juice,	Not declared
23	Kikura M	2004	RCT CO	RW	M+F	H	30 - 45	24	acute	WW	300 - 350	Not declared
24	Naissides M	2004	RCT CO	RW	F	Hypercholesterolaemia, postmenopausal	50 - 70	17	acute (3 times in 2 weeks)	DRW, W	400	National Heart Foundation of Australia
25	Whelan AP	2004	RCT CO	RW	M	CVD	30 - 70	14	acute	WW	4 mL/kg	Southland Medical Foundation, Invercargill, New Zealand.
26	Williams MJ	2004	RCT CO	RW	M	CVD	48 - 70	13	acute	WW	4 mL/kg	Not declared
27	Coimbra SR	2005	RCT	RW	M+F	hypercholesterolaemia	40 - 60	16	14 (x2)	purple grape juice	250	FAPESP and Fundação Zerbini.
28	Avellone G	2005	RCT CO	RW	M+F	H	35 - 65	48	28 (x2)	usual RW	250	Sicilian Agricultural Development Bureau, Palermo
29	Guarda E	2005	RCT	RW	M+F	CVD	55 - 62	20	60	W	250	Not declared
30	Hansen AS	2005	RCT	RW	M+F	H	38 - 74	69	28	red grape extract, W	M: 300 ml/day, 38.3 g ET/day, F: 200 ml/day, 25.5 g ET/day	Not declared
31	Karatzi KN	2005	RCT CO	RW	M+F	CVD	40 - 60	15	acute	DRW	250	Not declared
32	Pignatelli P	2005	RCT	RW	M+F	H	35 - 50	20	15	W, WW	300	Not declared
33	Retterstol L	2005	RCT CO	RW	M+F	H	40 - 60	87	21	W	150 (15 g ET)	Sigurd K. Thoresen Foundation
34	Tsang C	2005	RCT	RW	M+F	H	23 - 50	20	14	W	375	Not declared
35	Ziegler S	2005	RCT	RW	M+F	H	22 - 32	60	acute	WW	300	Jubiläumsfonds der Österreichischen Nationalbank
36	Zilkens RR	2005	RCT CO	RW	M	H	20 - 65	24	28	beer, DRW	375	National Health and Medical Research Council of Australia

37	Banini AE	2006	RCT	RW	M+F	T2DM, H	45 - 75	29	28	Dz-W, MJ, MW	150	College of Agriculture and Life Sciences
38	Beulens JW	2006	RCT CO	RW	M+F	H, WC>94cm	35 - 70	34	28	DRW	450 (40 g ETI)	Dutch Foundation for Alcohol Research
39	Blackhurst DM	2006	RCT	RW	M+F	H	25 - 45	15	acute	W	M: 230, F: 160	Wine Industry of South Africa
40	Boban M	2006	RCT CO	RW	M	H	25 - 40	9	acute	DRW, ET, PSRW, W	3 mL/kg BW	Not declared
41	Jensen T	2006	RCT CO	RW	M+F	H	35 - 70	92	21	W	150 (15 g ET)	Not declared
42	Marfella R	2006	RCT	RW	M+F	T2DM, MI	30 - 40	131	1 year	W	118 (11g ET)	Not declared
43	Naissides M	2006	RCT	RW	F	Hypercholesterolaemia postmenopausal	50 - 70	45	42	DRW, W	400 (40 g ET)	National Heart Foundation of Australia
44	Papamichael C	2006	RCT CO	RW	M+F	H, smokers	25 - 35	20	acute	DRW	250	Not declared
45	Addolorato G	2007	RCT	RW	M+F	H	20 - 30	30	30	beer, spirit	400; 11% ET	Association for Research in Medicine' Foundation Bologna-Rome(Italy).
46	Djurovic S	2007	RCT CO	RW	M+F	H	40 - 60	87	21	W	150 (15 g ET)	Not declared
47	Gorelik S	2007	RCT CO	RW	Not declared	H	25 - 35	10	acute	#	200	BARD, The United States-Israel Agricultural Research and Development Fund.
48	Karatzi K	2007	RCT CO	RW	M	Heavy smokers	22 - 24 and 66 - 75	12	acute	DRW, SMOKING	250	Korea Research Foundation and USDA/ARS Western Human Nutrition Research Center at the University of California at Davis.
49	Hijmering ML	2007	RCT	RW	M+F	H	25 - 45	20	acute	low-polyphenolic alcoholic fruit-flavored drink	≃ 330	Not declared
50	Micallef M	2007	RCT CO	RW	M+F	H	18 - 30	20	14	W	400	Not declared

51	Modun D	2007	RCT CO	RW	M	H	25 - 40	36	acute	DRW,ET, PSRW,W	195 – 280	Ministry of Science,Education and Sports of the Republic of Croatia
52	Sacanella E	2007	RCT CO	RW	F	H	20 - 50	35	28 (x2)	W, WW	200 (20 g ET)	–
53	Shai I	2007	multicenter RCT	RW	?	T2DM	41 - 74	91	12 weeks	non alcoholic beer, WW	150 (13g ET)	Not declared
54	Spaak J	2007	RCT	RW	M+F	H	24 - 47	13	14 (x3)	ET, W	155 (12% ET)	Heart and Stroke Foundation of Ontario and the Canadian Institutes of Health Research
55	Vázquez-Agell M	2007	RCT CO	Cava (sparkling wine)	M	H	25 - 43	20	28	gin	300 (30 g ET)	Spanish Ministries of Education and Science and Health
56	Gibson A	2008	RCT CO	RW	M	H	21 - 70	78	14	VDK	240 ml RW or 80 ml vodka (24 g ET)	The Pantridge Foundation; National Institutes of Health
57	Marinaccio L	2008	RCT	RW	M+F	stable CVD	50 - 70	45	acute	Gin, W	180 (18.9 g ET)	Not declared
58	Tousoulis D	2008	RCT	RW	M+F	H	22 - 27	83	acute	beer, whisky, W, WW	264	University of Athens.
59	Estruch R	2009	RCT CO	RW	M	H	30 - 50	40	28	gin	30 g/ET	Institutional
60	Nakamura T	2009	RCT	RW	M+F	T2DM with nephropath y	45 - 65	20	6 months	W, WW	118	Not declared
61	Huang PH	2010	RCT	RW	M+F	H	30 - 40	80	21	Beer, VDK, W	100	Institutional
62	Kaul S	2010	RCT	RW	M+F	H	30 - 50	12/11/11/11	14	VDK, W, WW	355; 59 ml VDK	National Institutes of Health
63	Kechagias S	2011	RCT CO	RW	M	high CVD risk	55 - 75	67	28	DRW, gin	30 g ET	Institutional
64	Kiviniemi TO	2010	RCT CO	RW	M	H	20 - 25	22	acute	DRW	120	Institutional
65	Cameli M	2011	RCT CO	RW	M+F	H	20 - 30	64	1	fruit juice	337.6± 68.9 (0.5 g ET/kg)	Not declared
66	Chiva-Blanch G	2011	RCT CO	RW	M	H	55 - 75	67	28	DRW, gin	30 g ET/d	Not declared
67	Chiva-Blanch G	2012	RCT CO	RW	M	high CVD risk	55 - 75	67	28	DRW, gin	30 g ET	Institutional

68	Noguer MA	2012	RCT CO	DRW	M+F	H	25 - 40	8	14	LPD	300 (+ low phenolic diet)	Institutional
69	Queipo-Ortuño MI	2012	RCT CO	RW	M	H	45 - 50	10	20	DRW, gin	272	Institutional
70	Schrieks IC	2012	RCT CO	RW	M	Overweight	35 - 68	19	30	DRW	450 (41.4 g ET)	Dutch Foundation for Alcohol Research
71	Barden AE	2013	RCT	RW	M	T2DM	20 - 65	25	acute	DRW, W	375 (41 g ET)	Not declared
72	Banach J	2013	RCT	RW	M	H	20 - 30	12/11/11/12/11	5	ET, black currant juice, W, WW	300	Collegium Medicum of The Nicolaus Copernicus University
73	Clemente-Postigo M	2013	RCT crossover	RW	M	H	45 - 50	10	20 days	DRW, gin	272	Institutional
74	Droste DW	2013	RCT	RW	M+F	Carotid Atherosclerosis	55 - 75	56/52	20 weeks	MD,W	F 100 ; M 200	Centre de Recherche Public-Santé
75	Kasicka-Jonderko A	2013	RCT	RW	M+F	H	21 - 32	12	1	Beer, W, whiskey	200 (13.7g ET)	Medical University of Silesia
76	Droste DW	2014	RCT	RW	M+F	Carotid Atherosclerosis	55 - 75	56/52	20 weeks	MD,W	F 100 ; M 200	Centre de Recherche Public-Santé
77	Muñoz-González I	2014	RCT	RW	M+F	H	20 - 65	34/8	28	W	250 1758 mg of gallic acid equivalents/L and 12% ET w	Institutional
78	Fantin F	2015	RCT	RW	M+F	H	25 - 53	18	1	/	300 (12% ET)	Not declared
79	Gepner Y	2015	RCT	RW	M+F	T2DM	50 - 65	27/27	6 months	W	150 (16.9 g ET)	European Association for the Study of Diabetes
80	Gepner Y	2015	RCT	RW	M+F	T2DM	50 - 70	224	2 years	W, WW	150	European Association for the Study of Diabetes
81	Moreno-Indias I	2015	RCT CO	RW	M	Obese, MeTs	45 - 50	10/10	30+30	DRW	RW or DRW: 272	Institutional
82	Mori TA	2015	RCT CO	RW	F	H	25 - 49	24	28 x 3	DRW, high RW, low RW	200 - 300 (146–218 g ET/wk)	National Heart Foundation of Australia.
83	Barroso E	2016	RCT	RW	M+F	H	**?**	15/26	28	W	200	Institutional

84	Golan R	2016	RCT	RW	M+F	T2DM	40 - 75	27/21	2 years	W	150	Institutional
85	Marhuenda J	2016	RCT CO	RW	F	H	18 - 27	9/9	37	RW (3 types)	200	Institutional
86	Mori TA	2016	RCT CO	RW	M+F	T2DM	49 - 66	24	28 (per period)	DRW, W	F: 230 (~24 g ET); M: 300 (~31 g ET)	Australian Health Management Group Medical Research Fund
87	Xanthopoulou MN	2016	RCT CO	RW	M	H	25 - 39	10	1	ET, W, WW	4 mL/ kg BW	Institutional
88	Chiu HF	2016	RCT	RW	?	H, hypercholes terolaemia	?	21	10 weeks	red onion extract	250	Taiwan Tobacco and Liquor Corporation (TTL),
89	Argyrou C	2017	RCT	RW	M	H	25 - 39	10	1 (x4)	WW, ET, W	4 mL/ kg BW	Graduate Program of the Department of Nutrition and Dietetics, Harokopio University.
90	Barden AE	2017	RCT CO	RW	M	H	20 - 65	22	28 (x3)	DRW, W	375	Institutional
91	Taborsky M	2017	RCT multicenter	RW	M+F	H	30 - 60	74/72	1 year	WW	200-300	Vino e Cuore, Ltd
92	Barden AE	2018	RCT CO	RW	M+F	T2DM	40 - 70	24	28 (x3)	DRW, No T2DM	W: 230 ml/day (~24 g ET/day) / M: 300 ml/day (~31 g ET/day),	Institutional
93	Di Renzo L	2018	RCT	RW	M+F	H	18 - 65	54	acute	HFM, MeDM, VDK, WW	30g ET	the Ministry of Agriculture, Italy
94	Golan R	2018	RCT (post hoc analysis)	RW	M+F	TD2M	50 - 70	117/57	2 years	W, WW	150: dry RW, (16.9 g ET), dry WW (15.8 g ET) or W	Institutional
95	Wotherspoon A	2020	RCT CO	RW	M	H	21 - 70	77	28	W	240	The Partridge Foundation
96	Roth I	2018	RCT CO	RW	M	high CVD risk	55 - 80	41	acute	Gin	AAW or gin (0.5 g ET/kg)	Fundación dela Investigación sobre Vinos y Nutricia
97	Fragopolou E	2021	RCT	RW	M	CAD	50 - 70	57	2 months	ET, W	200	Graduate Program of the Department of Nutrition and Dietetics, Har- okopio University and Hellenic Atherosclerosis society.
98	Briansó-Llort L	2022	RCT CO	RW	M+F	H	30 - 50	26	28	RW (2 different)	187	Instituto de Salud Car- los III

99	Haas EA	2022	RCT CO	RW	M	CAD	55 - 65	42	21	W	250 (5d/wk)	São Paulo Research Foundation and others
100	Choleva M	2022	RCT	RW	M+F	CAD	50 - 75	64	2 months	ET, W	200	Graduate Pro- gram of the Department of Nutrition and Dietetics, Harokopio University and the Hellenic Atherosclerosis Society.

Table S1 - Mean features of all papers

CAD coronary artery disease; CO crossover; CVD Cardiovascular disease; DRW dealcoholized red wine; Dz-W dealcoholized muscadine grape wine; ET ethanol; F Female; H Healthy; HFM high-fat meal; HPN hypertension, LPD Low phenolic diet; M Male; MD Mediterranean Diet; MeDM Mediterranean meal; MeTs Metabolic syndrome; MI myocardial infarction; MJ muscadine grape juice; MW muscadine grape wine; PSRW Polyphenols-stripped red wine; RCT randomised controlled trial; RW red wine; T2DM Type 2 diabetes mellitus; VDK Vodka; W water; WC Waist circumference; WW White wine;**#** A: 250 g turkey cutlets. W; B: soaked in RW after heating plus 200 ml of RW; C: soaked in RW prior to heating plus 200 ml of RW
