# Supplementary material for: Health Effects of Red Wine Consumption: A Narrative Review of an Issue That Still Deserves Debate
Source: Nutrients. 2023 Apr 16;15(8):1921. doi: 10.3390/nu15081921 (PMC10146095; doi:10.3390/nu15081921)
Supplement: Supplementary file 1 [file nutrients-15-01921-s001.zip › Table S2 - Mean Topics of all papers.pdf]

ref.	year	author	Antioxidant Effects	CVD	Coagulation and platelet function	Endothelial function	Hypertension	Immune Function/Inflammation	Arterial stiffness	Lipids and Homocysteine	Body weight, Body composition and Adipocytokines levels	MeTs T2DM	Gut microbiota and gastrointestinal tract
10	2000	Agewall S				*							
11	2000	Caccetta RA								*			
12	2000	Senault C								*			
13	2001	Caccetta R								*			
14	2001	Ceriello A	*		*							*	
15	2001	De Vries JH	*										
16	2001	Van der Gaag MS								*			
17	2002	Mansvelt EP	*		*								
18	2002	Foppa M					*						
19	2002	Watzl B						*					
20	2003	Mezzano D			*								
21	2003	Pignatelli P			*								
22	2003	Watzl B						*					
23	2004	Kikura M			*								
24	2004	Naissides M								*			
25	2004	Whelan AP				*							
26	2004	Williams MJ						*					
27	2005	Coimbra SR				*			*	*		*	
28	2005	Avellone G						*		*			
29	2005	Guarda E	*			*							
30	2005	Hansen AS								*			
31	2005	Karatzi KN					*		*				

32	2005	Pignatelli P	*									
33	2005	Retterstol L						*				
34	2005	Tsang C	*							*		
35	2005	Ziegler S								*		
36	2005	Zilkens RR				*	*					
37	2006	Banini AE								*	*	*
38	2006	Beulens JW									*	*
39	2006	Blackhurst DM	*							*		
40	2006	Boban M				*						
41	2006	Jensen T			*							
42	2006	Marfella R										*
43	2006	Naissides M				*						
44	2006	Papamichael C					*					
45	2007	Addolorato G	*									
46	2007	Djurovic S									*	
47	2007	Gorelik S								*		
48	2007	Karatzi K				*						
49	2007	Hijmering ML				*						
50	2007	Micallef M	*									
51	2007	Modun D	*									
52	2007	Sacanella E						*				
53	2007	Shai I										*
54	2007	Spaak J				*	*					
55	2007	Vázquez-Agell M						*				
56	2008	Gibson A	*						*			
57	2008	Marinaccio L		*								

58	2008	Tousoulis D			*	*		*					
59	2009	Estruch R	*							*			
60	2009	Nakamura T										*	
61	2010	Huang PH				*							
62	2010	Kaul S		*	*								
63	2011	Kechagias S								*			*
64	2010	Kiviniemi TO		*									
65	2011	Cameli M		*									
66	2011	Chiva-Blanch G					*	*					
67	2012	Chiva-Blanch G								*		*	
68	2012	Noguer MA	*										
69	2012	Queipo-Ortuño MI					*						*
70	2012	Schrieks IC	*										
71	2013	Barden AE				*	*						
72	2013	Banach J			*	*		*					
73	2013	Clemente-Postigo M											*
74	2013	Droste DW								*			
75	2013	Kasicka-Jonderko A											*
76	2014	Droste DW		*									
77	2014	Muñoz-González I						*					
78	2015	Fantin F					*		*				
79	2015	Gepner Y					*						
80	2015	Gepner Y										*	
81	2015	Moreno-Indias I										*	*
82	2015	Mori TA					*						

83	2016	Barroso E											*
84	2016	Golan R									*		
85	2016	Marhuenda J	*							*			
86	2016	Mori TA					*			*			
87	2016	Xanthopoulou MN			*								
88	2016	Chiu HF	*							*			
89	2017	Argyrou C			*								
90	2017	Barden AE	*			*	*	*					
91	2017	Taborsky M								*			
92	2018	Barden AE						*				*	
93	2018	Di Renzo L	*										
94	2018	Golan R		*									
95	2020	Wotherspoon A						*			*		
96	2018	Roth I		*									
97	2021	Fragopolou E						*					
98	2022	Briansó-Llort L								*	*	*	
99	2022	Haas EA											*
100	2022	Choleva M	*							*		*	

Table S2 - Mean Topics of all papers

* means that the paper addresses the relationship between red wine consumption and topic
