# Supplementary material for: Health Effects of Red Wine Consumption: A Narrative Review of an Issue That Still Deserves Debate
Source: Nutrients. 2023 Apr 16;15(8):1921. doi: 10.3390/nu15081921 (PMC10146095; doi:10.3390/nu15081921)
Supplement: Supplementary file 1 [file nutrients-15-01921-s001.zip › Table S3 - Long-term studies.pdf]

ref	year	author	Effects of RW consumption	Wine consumption duration	Type of Study	Patients	Age range	Total number of participants
60	2009	Nakamura T	renoprotective effect (RW, not WW)	6 months	RCT	T2DM with nephropathy	45-65	20
79	2015	Gepner Y	= mean 24-hour BP	6 months	RCT	T2DM	50-65	27+27
42	2006	Marfella R	↓ the increase in oxidative stress and inflammation	1 year	RCT	T2DM, MI	30-40	131
91	2017	Taborsky	RW = WW on lipid profile, CRP, fasting blood glucose, and HDL levels	1 year	RCT multicenter	H	30-60	74+72
80	2015	Gepner Y	↓ modestly CVD risk.	2 years	RCT	T2DM	50-70	224
84	2016	Golan R	= weight gain; = abdominal adiposity	2 years	RCT	T2DM	40-75	27+21
94	2018	Golan R	= carotid thromboembolism;<br>small ↓ plaque load in subjects with increased plaque load	2 years	RCT (post hoc analysis)	TD2M	50-70	117+57

Table S3 - Long-term studies

↓ reduced; = same; BP Blood Pressure; CRP C-reactive Protein); CO crossover; CRT C-reactive protein; CVD Cardiovascular disease; ET ethanol; H Healthy; HDL high-density lipoprotein RCT randomised controlled trial; RW Red Wine; T2DM Type 2 diabetes mellitus; WW White wine
